# The Destructive Loop: Dealing and Coping With Destructive Leadership

**DOI:** 10.1111/sjop.70076

**Published:** 2026-01-14

**Authors:** Maria Fors Brandebo, Miriam van Baalen

**Affiliations:** ^1^ Swedish Defence University Stockholm Sweden

**Keywords:** coping, destructive leadership, organizational culture, workplace silence, workplace stress

## Abstract

Previous research on destructive leadership has mostly focused on the destructive behaviors and outcomes and less attention has been paid to how to cope with and handle this kind of stressor. The overall aim of this study is to gain a deepened understanding of how subordinates and superiors react to (cope with and manage) destructive leadership behaviors and if and how organizational culture is perceived to influence the chosen strategies. This study used a grounded theory approach and is based on interviews with 26 individuals in the Swedish Armed Forces who had experience of a destructive leader, either as their superior or as their subordinate. The data represents both women and men, different ages, civilian and military backgrounds, and a variety of ranks and branches. The results suggest that the process surrounding being exposed to, coping with, or managing destructive leadership behavior, both from a subordinate and superior perspective, can be understood as *a destructive loop*. In this loop, individuals relate to, are constrained by, and participate in the coproduction and reproduction of environmental constraints present in the organization. Subordinates use mostly emotion‐focused strategies (such as withdrawal) when dealing with destructive leadership, while superiors use problem‐focused strategies (e.g., direct action against the leader). Environmental constraints (organizational, cultural, and norm‐related) function as enablers of the destructive behavior and barriers to effective coping−management strategies. By combining the perspectives of subordinates and superiors on destructive leadership, the authors add to the literature by painting a picture of how contextual aspects constrain constructive actions and strategies when dealing with the stress of being exposed to destructive leadership. *The destructive loop* highlights how destructive behavior can be coproduced and reproduced, placing the phenomenon of destructive leadership within a broader organizational hierarchy.

## KEY POINTS

- Contextual aspects can constrain strategies used to cope with and manage destructive leadership, coproducing and reproducing destructive behaviour rather than minimising or mitigating it.
- Superiors tend to use strategies that mostly can be categorized as problem‐focused, while, for subordinates, the pattern is the opposite: the strategies are mostly emotion‐focused.
- Negative organizational culture, such as culture of silence, can lead to superiors and subordinates not using constructive strategies when exposed to destructive leadership.

## 1 Introduction

Previous research on destructive leadership has mostly focused on destructive behaviors and outcomes, and less attention has been paid to how to cope with and handle this kind of stressor (Schyns and Schilling 2013). Exposure to destructive leadership has been described as one of the most emotional and disturbing experiences in the workplace (Matta et al. 2014; Simon et al. 2015), and something most individuals will experience during their working life (Aasland et al. 2010). Therefore, it is important to understand how to counteract the negative effects. Destructive leadership has been defined as “the systematic and repeated behaviour by a leader, supervisor or manager that violates the legitimate interest of the organisation by undermining and/or sabotaging the organisation's goals, tasks, resources and effectiveness and/or the motivation, well‐being or job satisfaction of subordinates” (Einarsen et al. 2007, 208). This definition is the point of departure for the current study, since it takes negative outcomes for (and differentiates between) both the subordinates and the organization into account, as well as both instrumental and emotional aspects. The definition also assumes that destructive behaviors happen repeatedly and not necessarily with negative intention; what counts are the consequences. The conceptual model proposed by Einarsen et al. (2007) describes four different destructive leadership types. The model also illustrates that there are two different dimensions that destructive leadership can be understood by: subordinate and organizational. Each dimension ranges from pro to anti depending on where the destructive behavior is directed. Tyrannical leadership is an anti‐subordinate and pro‐organization type, derailed leadership is anti both subordinate and organization, while supportive‐disloyal is a pro‐subordinate, anti‐organization type. Laissez faire‐leadership is placed in the middle of the dimensions. However, whether the last type is appropriately placed in the middle deserves further discussion, as research suggests that the passive forms of destructive leadership may be more harmful than the active forms, and thus might be better positioned in the same spot as derailed leadership.

Destructive leadership is often divided into active and passive forms. Active forms include behaviors such as arrogance, unfairness, threatening or punishing subordinates, while passive involve not showing an active interest, failing to confront others, or poor structuring and planning (Larsson et al. 2012). Active behaviors are often considered more deliberate and volitional (Einarsen et al. 2007), but it has been suggested that passive forms are most prevalent (Aasland et al. 2010; Lundmark et al. 2021), primarily used by leaders who have abdicated from their responsibilities and duties.

There are several parties involved when destructive leadership occurs in an organization. Usually the destructive leader, one or more affected subordinates, and one or more superiors who are hierarchically superior to the destructive leader. These parties' behaviors, attitudes, and feelings do not occur in a vacuum but are most likely influenced by the organizational culture and structures (Kusy and Holloway 2009). Although Padilla et al. (2007) introduced the “toxic triangle,” emphasizing the combined impact of leaders, subordinates, and their environment leading to destructive leadership and its negative outcomes, very few studies move beyond the leader's behavior and the outcome for employees. Environmental constraints have been suggested to determine coping‐strategies since it mitigates use of resources (Lazarus and Folkman 1984). For example, counterproductive work behavior (CWB) has been proposed to be a coping strategy that subordinates use when exposed to destructive leadership (Mawritz et al. 2014). Both CWB and destructive leadership have been linked to a more negative organizational culture which allows these behaviors to continue (Tepper 2007; Kusy and Holloway 2009; Mulvey and Padilla 2010). Another example is when subordinates use silence as a response to destructive leadership, allowing the destructive behavior to continue (Song et al. 2017). Based on the fact that there is not much knowledge about how individuals can cope with and manage destructive leadership, and what role the environment (organizational culture) plays in these processes, the overall aim of this study is to gain a deepened understanding of how subordinates and superiors react to (cope with and manage) destructive leadership behaviors and if and how organizational culture is perceived to influence the chosen strategies. The specific research questions are:

- What coping and management strategies do subordinates and superiors use in response to destructive leadership behaviors?
- How are the chosen coping and management strategies perceived to be influenced by the organizational culture and structures?

Behavioral and cognitive efforts to deal with external stressors (such as destructive leadership) can be divided into problem‐focused and emotion‐focused coping. Problem‐focused coping aims to try to change the problem that causes stress, while emotion‐focused coping aims to help the individual to manage his or her emotions toward the problem (Lazarus and Folkman 1984). Examples of the former are problem‐solving, information seeking, and communication. Emotion‐focused strategies include distancing, reappraisal, and seeking support. Coping strategies can also be sorted by the degree of confrontation (from low to high), where both types of strategies can vary in degree of confrontation. Strategies like avoidance are considered non‐confrontational (May et al. 2014). Drawing upon Lazarus and Folkman (1984), Yagil et al. (2011) developed a scale focusing on coping with toxic leadership. Five strategies were included: ingratiation, direct communication, avoidance of contact, support seeking and reframing. Webster et al. (2016) used the theoretical frameworks of Yagil et al. (2011) and Skinner et al. (2003) to categorize coping strategies reported by employees exposed to destructive leadership. They found support for all categories in the previous two mentioned studies: problem‐solving/direct communication, support seeking, escape/avoidance of contact, accommodation/reframing, information seeking, submission, self‐reliance, helplessness, delegation, and ingratiation. The most common strategies reported were support seeking, escape/avoidance of contact, submission, and problem‐solving/direct communication. All but the last one are considered emotion‐focused strategies.

However, when examining coping‐strategies used in response to destructive leadership behaviors, it is also essential to consider how functional these strategies are, and for whom. Coping‐strategies can be both functional and dysfunctional. The former implies that the individual and/or the organization benefits positively from the strategy (Ferguson and Cox 1997) while the latter means that the individual and/or the organization does not. Retaliation can, for example, make the subordinate feel better momentarily (creates a sense of justice) but is not functional for the organization since there is a risk of the situation escalating (Liang et al. 2022). Generally, problem‐focused strategies, for example, confrontation, have been suggested to be more functional since they deal with the problem itself (Yagil et al. 2011). Previous studies on coping and destructive leadership behavior show contradictory results regarding how functional they are. For instance, avoidance has been suggested to be a functional strategy, at least in the short run (Nandkeolyar et al. 2014) but has also been connected to exhaustion, negative affect, and intention to quit (Ogunfowora et al. 2021; Peltokorpi 2019; Whitman et al. 2014; Yagil et al. 2011). Conflicting results have been shown also for other coping‐strategies, such as direct communication (Yagil et al. 2011) and seeking support (Arain et al. 2020; Sarwar et al. 2022; Yagil et al. 2011). These studies also have a weakness in that they are usually only studying coping in relation to active forms of destructive leadership (most often abusive supervision, see for example Bowling and Michel 2011; Tepper et al. 2001). Passive forms of destructive leadership (Larsson et al. 2012), that might require different countermeasures, remain relatively unexplored. More research is needed to understand how subordinates can cope with different forms of destructive leadership in more functional ways.

While some studies have touched on supervisors' perceptions, there is a lack of detailed research into their management strategies and interventions in relation to destructive leadership. Some research suggests that the perception and management varies by hierarchy and positional power (see Kusy and Holloway 2009). Supervisors focus on a leader's job performance, often overlooking harm done to employees, while subordinates see the impacts on individuals and team dynamics (Ambrose and Ganegoda 2020). Further, organizations are less likely to intervene when destructive behavior targets individuals rather than organizational goals or when displaying CWB (Schyns and Schilling 2013). However, it is not always the case that superiors knowingly overlook destructive leaders. The latter can avoid scrutiny and detection since they act ingratiatingly and constructively toward superiors and are reaching targets. Their destructive behavior is only directed at their immediate subordinates (Gerpott and Van Quaquebeke 2022), which means that their superiors are unaware of their behavior, known as tyrannical leadership (Einarsen et al. 2007). Further research is not only needed to understand which strategies superiors use in response to destructive leadership, but also to understand how organizational factors influence management strategies and interventions, and to unravel the individual psychological processes that superiors go through, especially in terms of their perceptions of their “room for manoeuvre” and the contextual dynamics at play.

Organizational culture and norms have a great impact on how employees think and behave; can be considered a social control system for attitudes and behaviors (Chatman and O'Reilly 2016); and greatly shape leader‐follower relations (Thoroughgood et al. 2018). Hierarchical organizations, such as the military, are often dependent on loyalty, trust, and respect, leading to subordinates being less likely to identify and report destructive leadership (Erickson et al. 2015). When the organizational culture leads to fear of being punished, it also leads to fear of making mistakes and the subordinate would rather avoid taking risks than risk being punished (Erickson et al. 2015). This may risk creating a culture of silence, also known as organizational silence (Morrison and Milliken 2000). The culture of silence is one of the most known forms of negative culture. There are several reasons for individuals remaining silent, such as fear for punishment or that they gain something by it (going along to get along) (Kacmar and Carlson 1997). The connection between destructive leadership and employee silence has also been established (see e.g., Xu et al. 2015).

Another form of negative organizational culture is related to performance pressure. Subordinates feel pressured to raise their performance or risk facing consequences. When subordinates are pressured to perform more than they can handle, there is a risk that they start using unethical behavior as a form of self‐preservation (Mitchell et al. 2018).

## 2 Method

### 2.1 Participants

The selection of participants followed grounded theory guidelines and was governed by a desire to find informants at different hierarchical levels in the Swedish Armed Forces (SAF) representing women and men, different ages, civilian and military, and a variety of ranks and branches. Twenty‐six individuals, previously or currently employed by SAF, participated in the study (almost all were currently employed). To be included, informants needed to have experience of a destructive leader either as their superior or as a subordinate. The number of informants was determined by the criterion of conceptual saturation.

Eleven of the informants were women and 15 were men. Six contributed with experiences from the perspective of managing a destructive leader (superior); 18 from the perspective of coping with a destructive leader (subordinate); and two from the experience of both. The distribution between military and civilian personnel was 20 to 6. The average age of all informants was 49, which was approximately the same across managers and subordinates, and civilian and military personnel. Different ranks were represented, ranging from non‐commissioned to mid‐ and higher‐ranking officers.

Some informants had specific competencies in HR/health/safety/work environment, which presumably could contribute to a greater readiness to navigate an organization when experiencing situations of destructive leadership. Further, a few military informants had experience from deployment to international missions, where it is likely that contextual and different situational factors were at play, which could influence how to manage or cope with destructive leadership.

Informants were found through the authors' network. Military colleagues and collaborative partners forwarded information about the study to their networks with a call to contact the first author if interested in participating. In turn, informants also called on other colleagues to register their interest. As a result, a snowball sampling method was implemented.

### 2.2 Data Collection

Participants were treated according to the norms of the Swedish Ethical Review Authority. The project underwent ethical vetting by the Board (Protocol 2022‐01379‐01). Data were collected through interviews, following a prepared guide for each informant perspective (superior and/or subordinate). The interviews consisted of open‐ended questions and individually adapted follow‐up questions, first covering common themes such as background (age, position, rank etc.). For both categories, the questions concerned the informants' experiences of destructive leadership found in a superior or subordinate leader (Tell me about your experience with destructive leaders; Can you describe the type of behaviors that the leader(s) used?); their coping‐ or management strategies and the related motivations, thoughts and feelings (How did you react/act?); actions taken by the informant (What worked or did not work to handle the situation?); as well as the reactions and actions of the other parties involved throughout the course of events (how did the leader react when they found out about your experience?; How did it affect your relationship?; Was your closest supervisor aware of the situation and how did they react? How did the subordinate leader respond to your approach in handling the situation?).

The questions remained the same throughout the data collection. However, as core variables emerged, the authors asked the informants about these variables if they had not already addressed them. Specifically, whether they had experience with or could relate to them, and if so, in what way.

The informants expressed interest in participating by contacting the first author by e‐mail. They then received additional information regarding the study's aim, implications for the informant, and how personal and study data were handled. The informants also received a consent form that was returned to the authors in connection to the interview. Most of the interviews were conducted between April and June 2022 via Zoom or phone. A few additional interviews were conducted in August and September 2022. The interviews were recorded and generally lasted about 60–90 min.

### 2.3 Data Analysis

Some interviews were conducted, transcribed, and analyzed before additional interviews were conducted, which facilitated the emergence of core variables. The interviews were transcribed verbatim and analyzed according to the constant comparative method introduced by Glaser and Strauss (1967). As a first step, data was analyzed through open coping, line by line, to identify descriptions of actions, thought patterns, and feelings associated with the themes of the interview. Codes derived were formulated in words resembling those used by the informants. For example, the quote “I had a sounding board in a colleague and good friend who is in a totally different organisation, not near me” was coded as “Seeking/securing support.” All codes were then compared to verify their descriptive content and to confirm that they were based on the data. For this step, the first author analyzed the first interview and then the second author reviewed the results. For the second and third interviews, the roles were reversed, that is, the second author was responsible for the analyses, and the first author reviewed the results. This procedure ensured that the authors had a shared understanding of how the analysis should be carried out. For the remaining interviews, the second author analyzed the material requesting feedback along the way.

**FIGURE 1:**
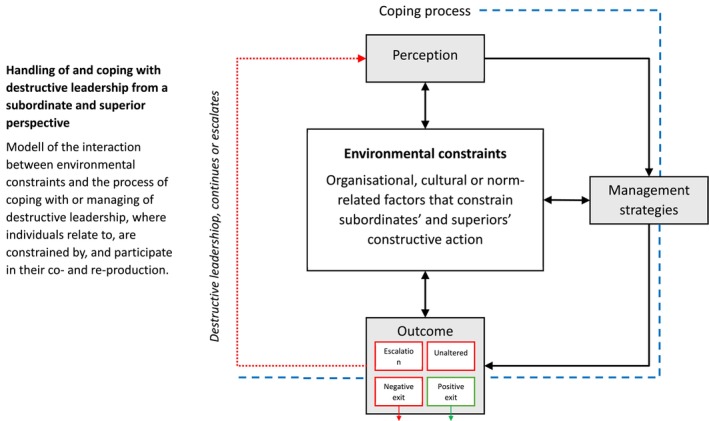
The destructive loop: handling and coping with destructive leadership from subordinate and superior perspectives. Model of the interaction between environmental constraints and the process of coping with or managing of destructive leadership, in which individuals relate to, are constrained by, and participate in its coproduction and reproduction.

As a second step, the codes were sorted into different categories. This was achieved by both authors, together, comparing interview transcriptions, codes and categories. Given the first author's prior experience in the research field, a self‐reflexive approach was employed. The second author had not, at this point, the same knowledge of the research field which ensured that both authors had to consider and express their assumptions. The example “code” mentioned above was sorted into the category “Superior leaders' strategies.” Ultimately, a large majority of the informants were represented in each category. This step was guided by asking the question “how do the informants respond to destructive leadership behaviours and what facilitates or hinders the success of their response.” The first author's background in work psychology naturally influenced the naming of the categories. Since both authors participated fully in step two and three, each code and category was discussed and motivated. The third step involved fitting the categories together using the constant comparative method, which in practice means that analysis steps were not strictly sequential; the authors moved back and forward, constantly re‐examining interview data, codes and categories. This resulted in four final core variables (Experience, Management, Outcome, and Environmental constraints). The example “Superior leaders' strategies” was sorted into the core variable “Management.” In this final step, colleagues of the authors (with extensive experience in research on leadership and/or coping and stress) were asked to discuss and comment on the proposed model (Figure 1). Minor adjustments to the model were made thereafter to clarify the process.

## 3 Results

In the following section, the destructive leader is referred to as “the leader” while the destructive leader's superior is referred to as “the superior leader” or “the superior.” Codes and categories are highlighted in italics.

### 3.1 The Destructive Loop

Based on the analysis, the process of being exposed to, coping with or managing destructive leadership behavior, both from a subordinate and superior perspective, can be described as *The destructive loop*. The results are presented in Figure 1. *The destructive loop* involves the interaction between environmental constraints and the process of coping with or managing destructive leadership. In the destructive loop, individuals relate to, are constrained by, and participate in the coproduction and reproduction of the environmental constraints that they perceive and experience. Regardless of whether the behavior is perceived as destructive, why and how it is detected, and what consequences it has, these environmental constraints influence all stages of the loop. They have an impact on the actions and coping strategies that are used to deal with the behavior, and subsequent outcomes. Thus, the individual and organizational processes at play can be understood against the backdrop of the destructive loop and the constraints. The strategies that subordinates and superiors perceive to be available, and their potential effectiveness, are greatly influenced by the organizational, cultural or norm‐related constraints identified. Further, the outcome of the initial actions/coping processes often lead to no change or an escalation of destructive behaviors, continuing the destructive loop. However, chosen strategies can also lead to a positive or negative exit. Below, the process is described in more detail, starting with perceptions of destructive leadership.

### 3.2 Experiencing and Discovering Destructive Leadership Behaviors

The destructive loop begins when a subordinate or superior catches the leader's behavior as destructive, that is, has a negative impact on the subordinate(s) (e.g., on their health) or the completion of tasks. Behaviors that were highlighted by the participants were *exclusion*; *self‐centredness; unreliability; unpleasantness; over‐controlling; passivity; lack of competence*; as well as *too task‐focused instead of relationship‐focused*.

### 3.3 The Management of and Coping With Destructive Leadership

#### 3.3.1 Superior Leaders' Strategies When Dealing With Destructive Leadership

This study identified several strategies used by superior leaders when dealing with perceived destructive leadership: *consequence assessment*, *delay‐action*, *gathering information*, *seeking/securing support*, *direct action against the leader*, and *taking subordinate‐directed action*. The results from this study show that being exposed to a destructive leader is not always as stressful for a superior as for a subordinate since the behavior is often directed at the latter. Therefore, the strategies superiors use are usually more problem‐focused.

*Consequence assessment* is a cognitive strategy, which includes leaning on previous experience and considering long‐term consequences. Here, previous experience from dealing with destructive leadership provides a basis for quickly identifying the problem to be dealt with and informs decision‐making about appropriate strategies. One superior leader explained:

There were some similarities with another [subordinate leader] I had last year. At that time, I was determined that the [unit] should deal with it [themselves] with support from me /…/ But that led nowhere. So, finally I had to step in there as well. And it was that experience that led me to take more firm action this time.

*Delay action* is a strategy that superiors often opt for or feel forced to do. It can be exemplified by giving the destructive leader a second chance and is motivated by a benevolent interpretation of the cause of the behavior (temporary, not the persons normal behavior), lack of replacement, or impression that the individual is about to leave anyway (e.g., retire). One superior explained that it is easier to remove someone during an international mission, because destructive leadership in that context means risking lives. Another reflected on how potential long‐term consequences played part in choosing the course of action:

No, I was not worried about vacancies. But I think that everyone deserves a second chance. We had [several talks]. And, with hindsight, I think too well of people, and that is a weakness sometimes. I do think that people can change, /…/ If someone says “Yes, I will do that [change]”, then you believe them.

*Gathering information* entails how superiors try to gather information and increase their presence to form an independent view after learning about the destructive behavior. It was stated that collecting documentation takes time. By being active and involved, superiors can receive first‐hand information and communicate directly with subordinates. It appears to be important to document meetings and chain of events, should it lead to a leader being removed.

The *seeking/securing support* category highlights the need for leaders to seek guidance and support from senior leaders, colleagues, or HR. Leaders benefit from both discussing issues and venting with peers or superiors, in addition to gathering information or feedback from subordinates. Superiors in the study had mixed experiences with HR support: some found it valuable, while others felt HR lacked necessary context and was hesitant to act. Seeking external support from other units with relevant experience or a coach to help process thoughts was also noted as beneficial. Receiving advice from or talking to an independent party, not entangled in the destructive culture, could provide perspective.

One last form of this strategy is to involve the destructive leader's deputy, which was said to be an effective way of dealing with the situation. In these cases, the deputy was informed and tasked to take on more responsibility and influence the destructive leader to change their behavior:

I took the deputy aside and gave [deputy] a clear task – “you have to make this work, I do not trust [destructive leader] 100%, [they] don't have the organisation's trust. You are trusted, you need to take greater responsibility and try to make the organisation work and make the subordinates happy”. Because [destructive leader] listened to [deputy] a lot. I used [deputy] as a lever to get a good outcome in the end. From experience, I know it works.

The category *direct action against the leader* primarily involves addressing relational issues through communication or coaching to fostering realization and behavior change. However, if the leader's destructive behavior creates an unworkable environment or refuses to acknowledge the problem and/or willingness to change, options may include transferring or removing them from their role. As termination is difficult, purposely reassigning them to an undesirable position can sometimes prompt resignation.

The final strategy for superior leaders, *taking subordinate‐directed action*, is applied when a destructive leader's behavior negatively impacts the working group or better communication is needed. Managing destructive behavior not only includes handling the leader but often also the group and the environment. Superior leaders may initiate discussions to drive improvement, though success is rare without openness to feedback and dialog. Direct action against subordinates can also take less constructive forms: sometimes, if the working group itself contributes to the destructive environment, superiors might unintentionally adopt destructive behaviors while attempting to resolve the situation. One superior reported: “During this period, as a result of my loss of trust in them, trust in my colleagues, I also started behaving destructively towards my personnel. /…/ I became more and more controlling, checking that they were following existing rules.”

#### 3.3.2 Subordinates' Coping‐Strategies When Dealing With Destructive Leadership

Several coping strategies used by subordinates have been identified in this study: *confrontation*, *indirectly solving the problem*, *shift of focus* (cognitive adjustment), *avoiding confrontation*, *seeking support*, and *withdrawal* (removing oneself from the problem). Most of the strategies are defined as emotion‐focused. Based on the participants' descriptions, it can be assumed that most individuals use multiple coping strategies and are deliberate in assessing the potential success rate before applying a strategy.

*Confrontation* involves openly expressing one's concerns to the destructive leader about how their behavior is perceived or found unacceptable. Typically, this coping strategy is followed by other strategies because confrontation alone is rarely successful. Most subordinates describe it as emotionally demanding and preceded by very careful consideration. The few describing confrontation as instant or spontaneous recalled it as a direct response in affect to an unacceptable situation.

Various factors influence the decision to confront a leader: a belief that the leader could potentially change in a positive direction; that they cannot negatively impact one's future career; anticipation of leaving the unit anyway; knowing that the leader will remain in charge; or a sense of responsibility to stand up for one's own subordinates:

I have experienced it before, when you have responsibility for someone, when I was the one leading, like being the boss of [unit]. Then it's easy to be assertive because I speak up for them. It was harder to stand up for myself.

Of those reporting that they had confronted a leader in some way, a majority were civilian employees.

The category *indirectly solving the problem* involves strategies without direct confrontation. Tactics include anticipating or circumventing the leader by pre‐emptively securing support for decisions or procedures through others, bypassing the leader, or proceeding without their input. It may also involve subtle communication, like seeking the leader's perspective, asking for guidance, or tactfully suggesting ways to handle tasks. To illustrate: “My method to try to make it better was, as always, to try to come up with constructive suggestions, “I have an idea” and I tried to plan it so that they could take credit for it.”

Subordinates may also secure evidence and gather documentation to support the removal of a destructive leader. Further, subordinates also described displays of subtle resistance, such as not laughing at the leader's jokes, leaving the room when they enter, minimizing effort, or avoiding interaction with the leader:

When [leader] came into the break room, I made sure I had things to see to. It was things like this that implicitly sent [leader] a signal, something I probably wanted to do. Because I didn't like what [leader] stood for and then we never spoke about it.

The category *shifting focus* (cognitive adjustment) involves strategies for mental distancing from a destructive leader by focusing on tasks or taking care of others; viewing the leader as temporary; or telling oneself that one is loyal to the task rather than the leader. However, these strategies often fall short, as they fail to address the root issue. Sometimes cognitive adjustment manifests as self‐examination, where subordinates consider or question their own part (examining feedback delivery, self‐blaming) instead of attributing problems to the leader's behavior:

I blamed myself for a long time. That I did something wrong, or that I was wrong, or that I had done something. /…/ I asked others “Did I say something wrong, did I do something wrong?”. I needed confirmation from others ‐ did anyone else observe this?

Shifting focus also involves positive thinking: hoping for change; showing compassion for the destructive leader by contemplating their perspective or connecting their behavior to external factors (such as the leader going through something difficult or not getting support from the organization); seeking positive role models; maintaining a positive outlook; or focusing on positive aspects of the job such as rewarding tasks. To illustrate: “I really made a 110% effort to be positive. “Yes, absolutely, I will take care of this” and so on. Just to try and meet [the leader] in such a way that they couldn't find anything negative.”

Thinking that the leader is going through a tough time or not getting sufficient support seems like a common strategy when the leader is using passive destructive behaviors.

One final example described how participants fantasize about hurting or taking revenge on the destructive leader. As there is no intent of acting upon these feelings, fantasizing is more a way of dealing with frustration that stems from active forms of destructive leadership. One subordinate participant said: “The number of times I have sat in meetings and seriously considered shooting [leader] in the head with my gun. That is huge, I can tell you. I have considered all kinds of painful deaths for [leader].”

In the category *avoiding confrontation*, subordinates actively choose to sidestep conflict with the destructive leader. This is the most common strategy, especially in relation to active destructive behaviors. Participants detail avoidance, endurance, or resignation as ways to cope, citing reasons such as being new to the unit; fear of conflict; duration (too late to solve the problem); or concerns about repercussions. Endurance was easier when daily encounters were limited:

There were several times when I came to work and when I passed the gates, I just bawled my eyes out. And I had to take a detour to avoid passing [them]. So, you create a life that allows you to stay, but you find strategies to avoid that person.

Several subordinates initially attempted confrontation but abandoned efforts after the leader had reacted negatively. Other reasons given for avoiding confrontation were the leader's minimal impact on everyday life, a lack of trust, perceived unreceptiveness, or skepticism about the potential for change/improvement:

No, I felt that I wouldn't be successful in this. I wouldn't do it because they is extremely careful never to make any formal mistakes. In that case, we would only have ended up playing some kind of blame game. They would have denied everything and required that I present some form of concrete evidence.

Further, subordinates might also refrain from confrontation because they fear punishment:

I could have bypassed [leader] and gone to my [superior leader]. That was a possibility. But then I think that I'd have been in a really, really difficult work situation. I'm afraid that [leader] would have put all time and energy into making life miserable for me.

*Seeking support* includes turning to various sources. When all subordinates face destructive behavior, they can find support and solidarity in shared experience amongst colleagues. However, if only one or a few are targeted, isolation may occur as colleagues avoid involvement to protect themselves. While discreet or covert support might be offered, it often feels insufficient due to its private nature, although there is understanding and empathy for self‐protection:

[Colleague] could say “I understand that this is hard, it is troubling”. /…/ But he never said anything in public. And to me it's worthless, when you can't stand up for your opinion. So, I actually felt sort of lonely and exposed during my whole [time] there.

This category also involves seeking organizational support by reaching out to more senior leaders, HR, or the union. Most subordinates reported a lack of such supportive responses, a reluctance to take responsibility or attempts to downplay the leader's behavior. When support was given, it appears not to have led to any change. Subordinates felt isolated, especially when the destructive leader held a top position, believing that management generally back each other, and that seeking support was futile. Overall, knowing where one could receive help and support from within the organization was difficult, albeit the need for support is apparent.

Lacking internal support, subordinates may turn to external sources such as health services, especially if the impact of destructive leadership has resulted in more severe health consequences such as PTSD or sick leave. Some sought support from other units, aiming to find a safe and affirming environment that boosted their sense of worth and competence, rebuilding their strength amid their struggles. One subordinate described it as “life changing”: “That in itself is tragic, that I need another context to feel competent and good and that I am worth being around /…/ that is tragic in itself.”

Several subordinates who sought support from superior leaders also described the destructive leaders' behavior as discriminatory, insulting, and as patronizing criticism. These behaviors are likely to be directed toward subordinates only and therefore difficult for superiors to discover. Despite reporting to superior leaders, all but one stated that no action was taken, citing various environmental constraints. Feeling disbelieved and unsupported, half the subordinates regretted confronting the destructive leader and seeking support.

The last coping‐strategy identified is *withdrawal*, meaning that subordinates try to remove or distance themselves from the destructive leader by asking for transfer or applying for a position elsewhere:

So I got transferred to another [unit]. I won't say it was lifesaving, but had I continued there [old unit] I'm not sure I'd have been here today. /…/ The risk is that I would have… No, it would have been even worse.

Despite the additional expenses (travel and accommodation) that seeking a new position could entail for some, it was deemed worthwhile to escape the challenging situation. However, requests for a transfer are often denied by the destructive leader, prompting some to apply for positions in different units, which the leader cannot prohibit or hinder.

### 3.4 Environmental Constraints

Environmental constraints emerge within the destructive loop, which are categorized into organizational and cultural, norm‐related factors. Participants considered these constraints as enablers of destructive leadership and barriers to their successful coping and/or management strategies. However, participants also considered these restraints to be connected to the outcome and influencing the perceptions of behavior as being destructive.

#### 3.4.1 Organizational Constraints

The organizational related constraints are a *lack of resources and time*, *inadequate recruitment and selection*, *lack of knowledge and discussion*, and *lack of action*.

Multiple subordinates (but only one superior leader) cite a *lack of resources and time* as reasons for the prevalence of destructive leadership. Challenging organizational demands leave little time for leaders to build trust, nurture relationships, and address conflicts, making it more difficult to create a culture characterized by openness and honest communication which is necessary for giving feedback. A superior leader explained:

In my experience, our company officers have a completely impossible task to carry out. And when you don't have time to talk to your subordinates/co‐workers, there is also no forum for feedback. /…/ I should spend way more [time myself] on building relationships with my subordinates. But there is a production demand that results in this falling by the wayside. And then we also fail to create a feedback culture.

Further, a subordinate described how lack of time affected their willingness to confront a leader:

Even if I had gone to my company officer, and he cared about it, he has a pile of other tasks on his desk. You could see that all the time. So, it was very difficult, feeling that you wanted to take up someone's time with your feedback or feedback from our working group /…/ [superior leaders] are already so overworked.

Both subordinates and superior leaders point to inadequate *recruitment and selection methods* as contributing to destructive leadership. Superiors often lack influence over appointments, meaning unsuitable candidates may still be promoted by others. Destructive leaders may also have strong references, and subordinates observe that such individuals can still advance despite senior leaders being aware of issues:

[Leader] is hotly debated, because there were discussions when they was going to be promoted. Should we really let [leader] through [to continued education]? But then [it was said] that they was so personally suited, and had been [position] and so forth. So, it was passed [the promotion].

This risks creating a sense of resignation, it is not worthwhile to try taking action since they observe that this type of leaders continues to advance in their careers. Subordinates risk encountering them in other situations. Both subordinates and superiors in this study share the impression of a *lack of knowledge* about destructive leadership and a dearth of discussion about the organization's desired leadership style. Individuals trying to raise awareness are often silenced and might give up trying:

[After participating in leadership courses, they] are so excited, stoked and willing, like ‘damn it, now we're going to do this for real’. Because they have been through the other [alternative] too. But they are subdued, silenced, muted, marginalised and so on.

Not talking about destructive leadership makes it harder to use constructive strategies (like direct communication) since it risks being a taboo issue to talk about.

Both subordinates and superiors observe a *lack of action* against destructive leaders, who are often given repeated chances despite known issues. Subordinates feel these leaders face few consequences, while superiors express frustration upon learning that others were aware yet did nothing. Reference checks are rarely conducted, according to both groups. One superior leader said: “These events that came up during the autumn, they had been known about for years when you started to unveil it. But nobody had really had the energy or courage to deal with it.”

Subordinates pointed out that destructive leadership behaviors may not be apparent to superior leaders because subordinates often “clean up” or mitigate the impacts, especially in case of passive leadership. This may lead to subordinates becoming stressed and exhausted. According to subordinates, superiors do not link high turnover or sick leave to destructive leadership. Even when the behavior is clearly visible, loyalty often prevents acknowledgement of issues. Action or recognition about the problem typically comes from leaders in other units, making it difficult for those within the unit to speak up, seek support or take action.

#### 3.4.2 Cultural and Norm‐Related Constraints

Cultural and norm‐related constraints can be divided into the categories *achievement culture*, *culture of silence*, and *socialization*.

The category *achievement culture* describes how the SAF is perceived to have a culture that emphasizes the rewarding and promoting of accomplishments over relationship‐building. This was described by both subordinates and superiors. Frequent officer rotations result in a prioritization and expectation of a high level of task performance to demonstrate competence for future advancement, for instance carrying out reorganizations. This focus on tasks, at the expense of relationships, is seen as a potential catalyst for destructive leadership. One subordinate explained:

But sometimes I believe that the CV becomes much more important than the ability to act. And that may be absolutely necessary in some situations, to be driven and maybe not too considerate, especially as war is our ultimate task. But when leading in peacetime /…/, I think it is disastrous.

An achievement culture influences the coping and management strategies in several ways. Superiors who try to use strategies such as direct action (transferring or removing the destructive leader) may be accused of ruining someone's career. A destructive leader can be successful in terms of results, and if the choice is between loosing a productive leader or making subordinates happy, the former may take priority. Almost all superior leaders in this study described how it is a difficult decision to remove or transfer a destructive leader from their position. Perceived to be a controversial course of action and often questioned, the superior needs to be prepared to explain and defend their decision. Even when the decision has been made, others (higher management, HR) can prevent its implementation:

But I think that [an external unit] contributed to [upper management] understanding the seriousness. /…/ we have not been correctly set up to discover and deal with destructive behaviours; we have let them go a little too far. Therefore, it was nice to have an external party come in and say “Hey! This is serious, this is unacceptable”. If we had dealt with it ourselves, I think there was a risk that we would have ended up thinking “Well, this isn't that bad”.

When the destructive leader's behavior is considered to be passive or lack of competence (passive forms of destructive leadership), an achievement culture can contribute to the leader being perceived to not be responsive to feedback since it may be disgraceful to not be decisive or portrayed as incompetent.

Additionally, a *culture of silence* is perceived to impact the detection and management of destructive leadership. The SAF is regarded to miss an active feedback culture, making subordinates hesitant about raising criticism or negative feedback. Silence is more common than seeking help or expressing concerns, as one superior described: “If you are brought up in our world, it is probably less common, I cannot imagine that you would bypass the destructive leader, you'd probably remain silent.”

Giving feedback or pointing out shortcomings, that is using a more constructive strategy as direct communication, becomes more challenging as you move up the hierarchy, according to both subordinates and superiors, who have observed a more pronounced culture of silence in higher positions:

Sometimes, as a platoon commander, you can be quite harsh towards a company commander. But, at my level, I'm not so sure that you would be that harsh with your battalion or unit commander. I'm unsure about that because, if I look at myself, when I was a company commander and was going to study to be a major or lieutenant colonel, you are in a position of dependency. And do you want to be a troublemaker, or do you want to be a silent follower?

This dependence on superiors, who may influence your future career, is a commonly expressed explanation for the culture of silence:

You are so often in a position of dependency vis‐à‐vis someone else [new]. You never know whether this person could soon be your boss, or on some board that will stop you from getting promoted /…/ Everyone knows everyone. /…/ It's always like “Oh, could this affect me soon? Will this person, even if I relocate or switch units or tasks, will this come back and bite me?”

Silence culture is also connected to fear, often expressed as respect for the hierarchy and loyalty to the organization. This hinders subordinates from confronting the leader. They believed it was inappropriate to question someone's fitness for a position and thereby the superior's decisions, considering the potential impact on promotion or career opportunities:

I think it is partly respect for the hierarchy. There is a leader who sits in a position that can affect me a lot. It is certainly cowardly of me, but it may to some extent depend on that. I am still dependent on these individuals.

One subordinate described finding it easier to avoid confrontation altogether, in view of the potential negative perceptions that could arise about themselves: “You easily become perceived as troublesome. Sensitive and troublesome. And who wants to work with that kind of person?”

Some speak of *socialization*, where certain units foster a specific culture that influences officer behavior and interaction, potentially promoting destructive leadership. One superior explained how the influence of constructive leaders tends not to spread to the same degree as the influence of destructive leaders:

And the interesting part is that we have an officer who is a real role model in how they practise developmental leadership to great effect. There are never any problems, they have very low turnover. People want to work with [leader] for years. And you might think that this could somehow become infectious, that others would see how they work and reflect “What does they do that makes things work so well?” and then start to copy [leader]. But this proliferation never happens in any way that makes an impact. /…/ And it is interesting that these more destructive leaders actually have much more impact than the developmental leader.

### 3.5 Outcome of Subordinates' and Superior Leaders' Coping Strategies

The interaction between environmental constraints and the process of coping with or managing destructive behaviors often leads to the coproduction or reproduction of these very behaviors. Strategies employed by subordinates and superior leaders vary in success, with only a few deemed effective. Outcomes include *escalation*, *unaltered situation*, *negative exit*, and *positive exit*. The first two imply that the destructive behavior continues or even increases, while the latter two may help break the destructive loop.

*Escalation* mainly occurs when reporting the problem or giving feedback directly to the leader exacerbates the situation. Seeking support may also lead to escalation, as subordinates are depicted as troublesome. Superiors described how the destructive leader may punish their subordinates for reporting to the superior leader. To illustrate:

What I've discovered is significant and recurrent. When I have had conversations with [leader], which the subordinates knew nothing about, they have been on the receiving end of retaliation. They have been threatened, they have been reprimanded. /…/ The retaliation occurred the following day or week. And it's just horrible.

Punishments can include minimal or no pay rise, being transferred against one's will, removal of responsibilities, or social exclusion:

I struggled and struggled, /…/ in the last year I received no e‐mails, I was not called to meetings. I was responsible for [tasks] on paper, but I was never allowed to participate in any meetings. /…/ I never received any invitations; I was never included.

Destructive leaders may isolate subordinates by prohibiting them from bringing support to meetings or limiting their contact with or access to certain individuals. Punishments may extend to colleagues who support these subordinates, impacting their work as well: “[Deputy] was not allowed to talk to me anymore, it should all go through [destructive leader]. /…/ I guess it was because I was questioning things.”

Destructive leaders may also punish through retaliation, by shifting perspectives of others and portraying the subordinate as disloyal, difficult, or problematic following confrontation. This portrayal can in turn prompt negative reactions and behaviors from subordinates, reinforcing the leaders' depiction and creating a cycle of increased anger and sensitivity. All this leads to subordinates refraining from using more constructive strategies, such as direct communication or seeking support, out of fear of facing punishment and leading to reinforcing a culture of silence.

Superiors can also experience punishment. The leader may attempt to turn the group against the superior by speaking negatively about them or instigating division, portraying the superior as the destructive one:

I know that before [destructive leader] quit, [destructive leader], frankly speaking, talked rubbish about both me and my [unit]. How bad everything was and so on. And of course, I could simply have replied by telling everyone what [destructive leader] had done. But then again, I'd have been betraying a principle that I hold very dear. So, I have to have faith in people being able to differentiate between bullshit and fact. But of course, it affects you when you are unable to or choose not to respond to that kind of criticism.

Subordinates perceive escalation not only when confronting destructive leaders, but also when seeking support from superiors; they become “the problem” that requires formal or informal intervention or action. One subordinate described how they were treated:

When I started at [new unit], I understood there was a discussion about me that depicted me as an inconvenience, a concern. And it started when I reported my boss for [harassment]. /…/ Because he [superior leader] then told me ‘you don't do this [report the leader], I didn't think you were that kind of person’.

This as well refrains subordinates from seeking support from the organization and contributes to the consolidation of the culture of silence. Approximately half the subordinates in this study expressed regret about confronting a leader or seeking support, advising others to avoid such actions as they often yielded no positive results. This was particularly true for those who described the destructive behavior as discriminating and/or domineering, who were more often civilians and/or women.

As mentioned earlier, superiors may adopt destructive behaviors, especially when they perceive the working group to play part in a destructive environment. They may fail to use constructive methods to solve the problem and instead become part of the problem, causing the situation to escalate (coproducing and reproducing destructive behavior).

This study reveals several instances where the strategies chosen by both superior leaders and subordinates fail to bring significant change—destructive behaviors remain *unaltered*. The leader can sometimes admit to destructive behavior but persists in exhibiting them, or becomes defensive or unwilling to understand feedback:

So I sit down with [leader] and we talk about this. And then comes this defence mechanism, because [leader] must explain him/herself. And it's everyone else's fault, not theirs. And they don't understand, those subordinates. They are too stupid and poorly selected. /…/ No understanding for the subordinates' circumstances.

A common outcome is the continuation of the destructive loop, even when the leader leaves, as a replacement may behave similarly or subordinates expect similar patterns. Leadership demands, like limited time or resources, might prompt negative behavior. Transferring or promoting the leader often fails to resolve the issue, merely shifting or escalating the problem.

Subordinates may perceive the superior leader to be destructive due to lack of information about how they deal with the destructive leader. The superior is viewed as passive and the situation remains unchanged. Managing these issues behind closed doors without informing subordinates, sometimes to protect the integrity of the parties involved, may be interpreted as inaction:

Personnel matters are problematic because I value the personal integrity and confidentiality of these individuals. Usually, I value that more than information dissemination. But it can lead to other subordinates feeling frustrated when they understand that something has happened – but they don't know what. /…/ some of them have… they perceive it as if we have covered things up.

When subordinates lack information regarding how, or even whether, the destructive leader is being addressed, the absence of transparency fosters a sense of hopelessness and discourages them from attempting to improve the situation.

The results show several examples of *negative exit* from the destructive loop, where subordinates and/or leaders leave the organization. While a subordinate leaving may be positive for their personal well‐being, it results in the loss of valuable competence. The negative exit may have long‐term consequences for both the subordinate and/or the destructive leader. The leader may face challenges in their career by being branded a “bad leader.” Subordinates may gain a bad reputation, which affects them in their new workplaces. Superiors who decided to remove a destructive leader described feeling guilty. The ousted leader may also hold a grudge for a long time, resorting to formal complaints, slander or vilifying the superior, attempting to tarnish the superior's reputation, or explicitly distancing themselves. To illustrate:

I told [leader] ‘You are welcome to stay in my [unit], but maybe in another [department] for support and development’. But we realised quickly that in some way I had become the symbol for everything that was bad with [leader's] career.

Many subordinates reported ongoing health issues and negative changes in their work behavior (such as withdrawal, not sharing opinions) after leaving their workplace. At the time of their interviews, some were still on sick leave; a few had been diagnosed with PTSD because of the experience. One subordinate described how s(he) is still affected:

I was signed off on sick leave. I was actually really ill. And I lost my job and like, my faith in life and the profession and the work and the Armed Forces and co‐workers and well, you know. I thought I was in the right place in life. /…/ I was a hair's breadth away from committing suicide.

A *positive exit* occurs when an individual or group manage to break out of the destructive loop. A subordinate leaving the unit may experience improved well‐being (content and happy with the new situation/position, even feeling empowered by pulling through). While positive on an individual level, on an organizational level it might imply losing competence or the continuation of destructive behavior now directed at others. Alternatively, and rarely, positive exits can involve a destructive leader changing their behavior after feedback, leading to improved relationships and termination of the destructive loop, or improved behavior after leaving for other positions.

The superior leaders in this study who have observed positive behavioral change in destructive leaders relate this to developing consciousness of one's behavior and its impacts. A superior recounted the reaction when speaking to a destructive leader about their behavior, after their initial defensive response over their negative attitude toward subordinates:

[Leader said] ‘I realise that I have been a destructive leader’. And I think that [leader] is the only one. /…/ who has managed to come to that realisation. And [leader] has made a complete U‐turn. /…/ I think [leader] will have a really strong inner compass about what is right and wrong going forward.

A positive exit can also occur through replacement, with a new and more constructive leader which benefits subordinates, superiors, and the organization. One superior said:

They [subordinates and destructive leader] tried to avoid each other as much as possible. That wasn't good either. But then [destructive leader] retired and we could recruit this new amazing leader. /…/ Here you see evidence that leadership can matter, when I think about it now. With the turnaround that came with the new boss, and the joy, there is only praise everywhere and still a very modest leader /…/ but also a very good leader.

Making a positive exit also involves instances where both the destructive leader and the environment/culture is addressed, minimizing the risk of further destructive leadership developing:

In [x] months, the [unit] was fixed. A combination of good leadership and a heavy hand. A number of [subordinates] who were in the inner circle, didn't yield or try to ingratiate themselves, but they were cleared out as well. They made a clean sweep in what was a rotten environment. And in [x] months they had turned the [unit] around. The problem was solved.

## 4 Discussion

This study investigates how subordinates and superior leaders cope with and manage a destructive leader in a military context, offering a unique combination of both perspectives. The study also includes organizational culture and studies if it is perceived to influence the chosen strategies. Previous studies have mostly been theoretical or have focused on leaders and outcomes, rather than including followers and environments in the equation (Thoroughgood et al. 2018). In this study, we place the phenomenon of destructive leadership within a broader organizational hierarchy. The study's findings are encapsulated in the “Destructive loop” (Figure 1).

The study identifies several coping strategies used by subordinates and superiors in dealing with destructive leadership. The strategies used by subordinates are consistent with those suggested by other studies (Webster et al. 2016). The primary contribution in the present study lies in painting a picture of how contextual aspects can constrain these strategies, coproducing and reproducing destructive behavior rather than minimizing or mitigating it. The superiors identified strategies that can mostly be categorized as problem‐focused, aimed at trying to minimize or prevent the destructive behavior and its negative consequences. For subordinates, the pattern was the opposite: the strategies are mostly emotion‐focused. This may reflect that subordinates feel they lack the power or position to influence the situation, leaving them with only the option of attempting to manage their own emotions, which is in line with previous studies (Webster et al. 2016). The military context is described as characterized by a culture of silence and achievement, which hinders constructive action. It has been suggested that problem‐focused coping, such as confrontation, is more effective because it aims to manage the stressor itself (Yagil et al. 2011). However, this study highlights that neither giving feedback nor seeking support from superiors is effective due to the prevalent culture of silence in the military, where the questioning of leaders is discouraged. Obedience is desirable in military officers and a characteristic that leads to trust in subordinate leaders (Fors Brandebo et al. 2013). Therefore, it can be assumed that individuals are socialized into silence (i.e., being obedient and loyal). This implies that context has a significant impact on the effectiveness, or otherwise, of coping strategies.

Some have proposed that destructive leadership and CWB might be a reflection of a more general negative organizational culture, where the organization is permissive or allows these behaviors to continue (Tepper 2007; Kusy and Holloway 2009). This study suggests not only that the organizational culture permits such behavior, but also that organizational factors, such as resource constraints and inadequate recruitment, might also drive these behaviors. Others have found similar patterns in a military context, where subordinates hesitate to report destructive leadership because of low levels of trust, a masculine culture, hierarchical barriers, loyalty and cohesion, and fear of exclusion (Dutch Central Government Audit Services 2023). Another study of destructive leadership in crisis management emphasizes the impact of a task‐focused context and achievement culture (Fors Brandebo 2020). When an achievement culture is perceived to prevail, the risk is that the organization allows destructive leadership to continue since taking measures might risk affecting productivity negatively, at least in the short term. Speaking in terms of the definition by Einarsen et al. (2007), as long as the destructive behaviors are not directed at or violate the legitimate interests of the organization, but are directed toward subordinates, the organization does not interfere. In sum, this underscores the importance of considering contextual aspects when studying and addressing destructive leadership.

The authors of this paper were hoping to find examples of functional strategies to deal with destructive leadership. Dishearteningly, the results show few, if any, strategies effective both to the subordinate and the organization. Subordinates' challenges may relate to positional power, in line with Kusy and Holloway's (2009) findings. Although superiors possess formal authority, this study reveals that using confrontational coping strategies against a destructive leader can lead to escalation of the situation. This likely reinforces the culture of silence and discourages effective strategies.

The *destructive loop* illustrates how destructive behavior can be reproduced. How many subordinates labeled as engaging in CWB may, in fact, be victims of destructive leadership? The results suggest that subordinates' chosen strategies are not only ineffective but may also be considered destructive to the organization (e.g., withdrawal). Those who challenge destructive leaders may be seen as disruptive, while superiors can be viewed as destructive if they appear passive or are misrepresented by retaliatory leaders. Consequently, even when a superior intervenes, they risk being labeled as destructive. Both subordinates and superiors sometimes adopt destructive behaviors themselves, often leading to escalation and furthering the destructive loop.

This proposition contributes to literature grounded in the conservation of resources theory, suggesting that when resources are stretched or depleted, individuals adopt defensive modes to preserve them. These can manifest as both active and passive destructive behaviors, with crucial resources including objects, personal characteristics, conditions, and energies. In a workplace context, these resources could include having a personal office, a sense of worth, job security, or work‐life balance. The actual or potential loss of these resources, despite investment, may trigger defensive strategies, leading to CWB (see Tafvelin et al. 2022 for a summary). Interestingly, both our participant groups attribute their experiences and the leaders' behavior to organizational, cultural, and normative factors linked to resources.

Effectiveness of coping strategies seems to be intrinsically linked to perceptions of organizational factors. To find effective coping strategies, addressing the organizational culture is crucial, especially if it facilitates destructive leadership and hinders effective management. A leader's behavior can be viewed as destructive or constructive in different situations or contexts (Fors Brandebo 2020). Similarly, coping‐strategies' effectiveness might vary based on the context in which the subordinate and the leader operate.

### 4.1 Practical Implications

Gaining knowledge and a thorough understanding of destructive leadership equips everyone in the organization, including exposed subordinates, superiors, and organizational representatives such as HR, with a shared vocabulary. This facilitates describing experiences in universally comprehensible terms, potentially mitigating mistrust between leaders and subordinates. To escape the destructive loop, efforts can focus on changing or influencing negative expectations. Understanding potential reactions to destructive leadership fosters self‐awareness, helping individuals to refrain from contributing to worsened situations. Organizational representatives, such as HR, can benefit from knowledge of the destructive loop, emphasizing the need for swift action. Clear routines for both subordinates and superiors are crucial because this study indicates that both groups often feel that they are left to handle the situation on their own.

Previous research shows that organizations are more inclined to act when the destructive behavior is directed at the organization than they are when the behavior targets subordinates (Krasikova et al. 2013). Our findings indicate that superior leaders typically discover destructive behavior when it is directed at the organization (e.g., security risk, resource misuse, lack of structure). It appears to be more difficult to detect destructive behavior directed at subordinates, allowing it to continue. From a superior's perspective, it can come down to the (destructive) leader's word against that of their subordinates. Gathering more information, especially first‐hand experience, takes time. Learning to recognize tyrannical leadership, where destructive behavior is aimed solely at subordinates, is crucial for superior leaders, as upper management may inadvertently view such behavior favorably (Einarsen et al. 2007).

### 4.2 Research Limitations

One limitation of this study is the selection method (snowball sampling), likely capturing individuals with more severe experiences. This may contribute to the scarcity of functional coping strategies, since those with successful initial strategies may not have perceived behavior as destructive enough, or the situation was resolved quickly. Additionally, the study's limited number of informants poses challenges for generalisability. Although theoretical saturation was used, further research is necessary to assess the representativeness of results.

### 4.3 Future Studies

We suggest that future studies continue to explore the interplay of factors and processes involving subordinates, superiors, and the organizational environment surrounding destructive leadership. Investigation of the connections between perceived organizational culture, coping strategies, and hierarchy levels is crucial. Additionally, further comparative studies are needed across different organizations to assess the universality or specificity of the cultural constraints found here. Furthermore, there should be continued research into identifying functional coping strategies and their effectiveness in various contexts and circumstances.

## 5 Conclusions

The present study indicates that subordinates predominantly rely on emotion‐focused coping strategies when exposed to destructive leadership. Superiors, on the other hand, appear more inclined to use problem‐focused strategies. Unfortunately, few of the identified strategies appear to be effective in minimizing destructive behavior, which may be linked to environmental constraints. The results suggest that the process of being exposed to, coping with, or managing destructive leadership, whether from a subordinate or superior perspective, can be understood as *a destructive loop*. In this loop, individuals relate to, are constrained by, and participate in the coproduction and reproduction of environmental constraints present in the organization. These contextual factors constrain constructive actions and strategies when dealing with the stress of being exposed to destructive leadership. More research is needed to understand how subordinates and superiors can manage destructive leadership more effectively.

## Author Contributions

**Maria Fors Brandebo:** conceptualization, methodology, investigation, formal analysis, writing, project administration. **Miriam van Baalen:** formal analysis, writing.

## Funding

This work was supported by Försvarsmakten.

## Conflicts of Interest

The authors declare no conflicts of interest.

## Data Availability

Data available on request due to privacy/ethical restrictions.
